# Halimanes and cancer: *ent*-halimic acid as a starting material for the synthesis of antitumor drugs

**DOI:** 10.3389/fchem.2023.1225355

**Published:** 2023-08-22

**Authors:** Alejandro M. Roncero, Ignacio E. Tobal, Rosalina F. Moro, David Diez, Isidro S. Marcos

**Affiliations:** Departamento de Química Orgánica, Facultad de Ciencias Químicas, Universidad de Salamanca, Salamanca, Spain

**Keywords:** halimanes, *ent*-halimic acid, antitumor, diterpenes, cancer, natural products

## Abstract

The development of new anti-cancer agents is an urgent necessity nowadays, as it is one of the major causes of mortality worldwide. Many drugs currently used are derived from natural products. Halimanes are a class of bicyclic diterpenoids present in various plants and microorganisms. Many of them exhibit biological activities such as antitumor, antimicrobial, or anti-inflammatory. Among them, *ent*-halimic acid is an easily accessible compound, in large quantities, from the ethyl acetate extract of the plant *Halimium viscosum*, and it has been used as a starting material in a number of bioactive molecules. In this work, we review all the natural halimanes with antitumor and related activities until date as well as the synthesis of antitumor compounds using *ent*-halimic acid as a starting material.

## 1 Introduction

Natural products (NPs) constitute an abundant and diverse source of chemical structures, which are currently used in the search of bioactive molecules and drug discovery (Karageorgis et al., 2021). In this manner, the study of natural products and their derivatives remains one of the most important research areas in organic, biological, and medicinal chemistry (Kumar and Waldmann, 2009; Karageorgis et al., 2021).

Among the last decades, natural product-like drugs have a higher success rate in showing bioactivity (Newman and Cragg, 2012). In fact, approximately half of the drugs in clinical use come from living organisms (Paterson and Anderson, 2005), and in the last few years, 73% of antitumor drugs were either natural products, bioinspired compounds, or natural product derivatives (Wilson and Danishefsky, 2006; Newman and Cragg, 2007).

This elevated bioactivity of NPs can be explained by the purpose of their biosynthesis itself (Danishefsky, 2010). Living organisms synthesize compounds in order to use them in their own metabolic pathways. Thus, these compounds fit in different kinds of proteins and enzymes involved in metabolic engineering. Hence, these compounds possess well-defined tridimensional structures rich in functional groups and adequately oriented in space, which can be used in drug modeling with a higher success rate.

Because of this, organic chemists have developed a number of strategies to obtain bioactive compounds inspired in natural products. These strategies consist of diversity-oriented synthesis (DOS), biology-oriented synthesis (BIOS), diverted total synthesis (DTS), analog-oriented synthesis (AOS), two-phase synthesis, function-oriented synthesis (FOS), computed affinity/dynamically ordered retrosynthesis (CANDOR), and more recently, a pharmacophore-directed retrosynthesis (PDR) (Wilson and Danishefsky, 2006; Truax and Romo, 2020).

Bioactive compounds are often asymmetric, and their biological activity is closely related to one of the enantiomers. However, there is also the possibility that both enantiomers show different activity. For this reason, the effective synthesis of homochiral molecules that are enantiomerically pure remains one of the biggest challenges that modern organic chemistry must face. Thus, in order to reach enantiomerically pure compounds, different methodologies can be applied from the kinetic resolution of racemic mixtures to the enantioselective synthesis using chiral natural products as starting materials (chiral pool) as well as the asymmetric synthesis using chiral auxiliaries, reagents, or catalysts for that purpose.

The synthetic approach to bioactive compounds by the transformation of easily accessible and abundant natural products (chiral pool) is widespread. The use of these natural products in the enantioselective synthesis of compounds of similar carbon backbones usually represent an advantage compared with the total synthesis in terms of economy of the process and synthetic steps. Many examples can be collected from the literature where chiral pools are used, but the examples of the synthesis of paclitaxel (Taxol^®^) (Gennari et al., 1996; Kingston et al., 2002) and ecteinascidin 743 (ET-743, Yondelis^®^) (Cuevas et al., 2000) are paradigmatic of this strategy.

**FIGURE 1:**
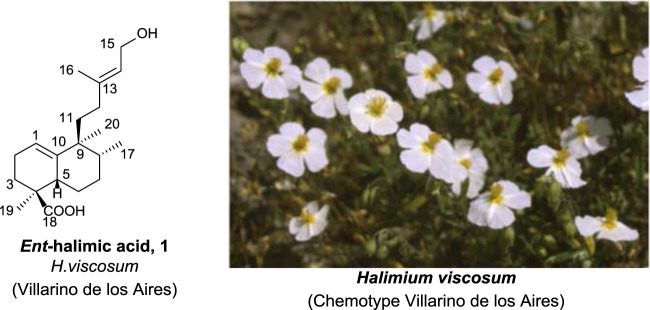
*Halimium viscosum* (chemotype Villarino de los Aires).

*Ent-*halimic acid **1** belongs to the bicyclic diterpene family of halimanes (Urones et al., 1987; Roncero et al., 2018). The use of **1** as a starting material allowed access to a variety of compounds with biological interest, such as antibiotic, antifeedant, antitumor, antifouling, and antiviral.. In this work, the use of *ent*-halimic acid **1** as a starting material in the synthesis of antitumor compounds is reviewed (Figure 1). Compound **1** is easily accessible in large quantities from the ethyl acetate extract of *Halimium viscosum* (*Cistaceae*). The vegetal source is widespread in the Iberian Peninsula, mainly in Spain (De Pascual Teresa et al., 1985; De Pascual Teresa et al., 1986; Urones et al., 1987) and Portugal (Rodilla et al., 1998; Rodilla et al., 2001).

The functionalization appearing in *ent-*halimic acid, with an allylic hydroxylic group in the side-chain and a Δ^1(10)^ double bond in the bicyclic system as well as a carboxylic acid at C-18, confer to excellent characteristics of (**1)** for its use as a starting material in the synthesis of antitumor compounds.

We have divided this work in two main aspects: first, we will summarize all the known natural halimanes, which exhibit antitumor as well as antitumorigenic-related bioactivities, and finally, we will review all the synthesis of bioactive compounds using *ent*-halimic acid as a starting material.

## 2 Natural bioactive halimanes

**FIGURE 2:**
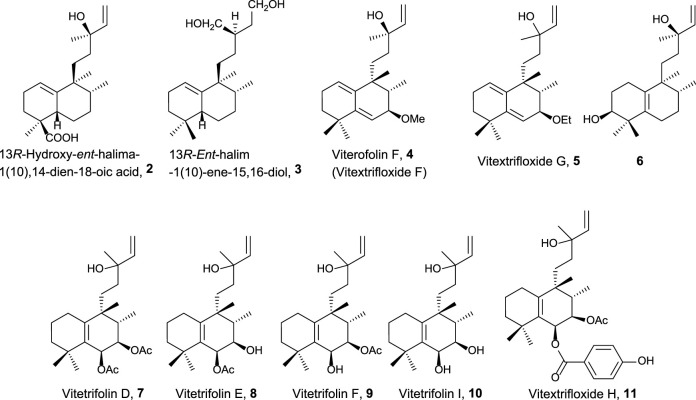
Halimanes showing an acyclic side-chain framework with antitumor and related activities.

**FIGURE 3:**
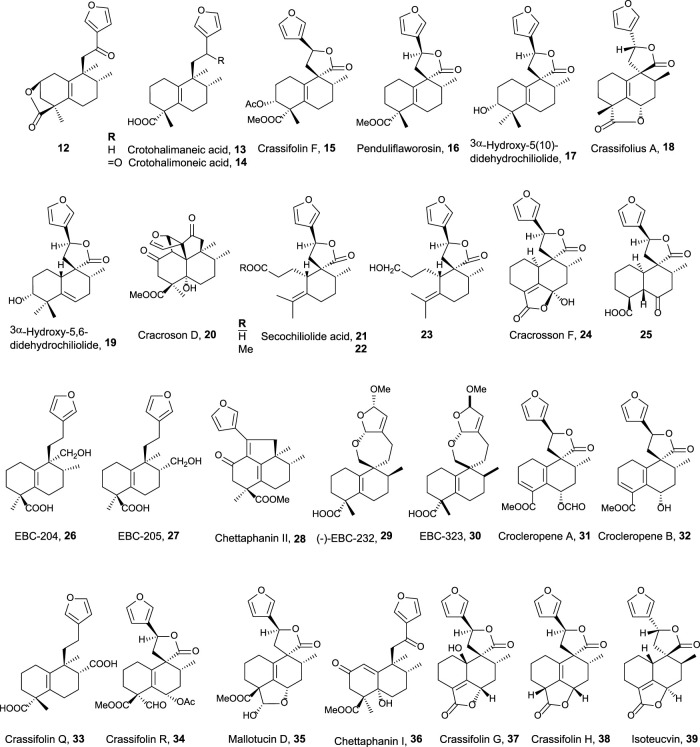
15,16-Furo-halimanes with antitumor and related activities.

**FIGURE 4:**
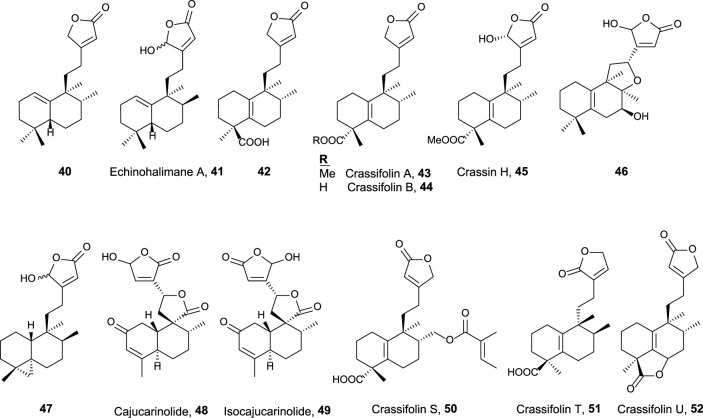
15,16-Halimanolides with antitumor and related activities.

**FIGURE 5:**
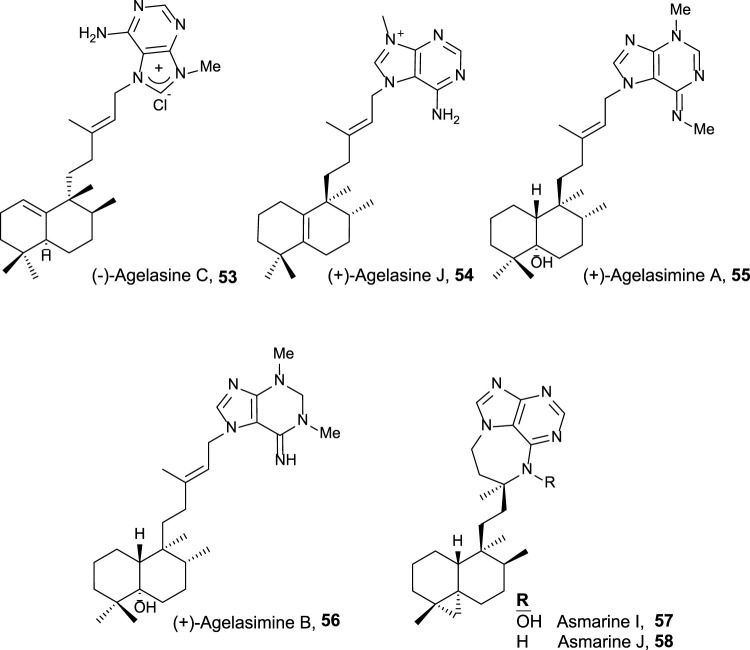
Halimano purines with antitumor and related activities.

Natural halimane skeleton compounds showing antitumor activity as well as antitumorigenic-related bioactivities are reviewed herein (Figures 2–5). These antitumor compounds have been classified into four groups according to their structural frameworks (data shown in parentheses represent the IC_50_ value of the corresponding compound).

The first group of natural halimanes is characterized by having an acyclic side chain (Figure 2), and all of them were tested *in vitro*, showing the following results:

• 13*R*-Hydroxy-*ent*-halima-1(10),14-dien-18-oic acid **2** showed low activity against the A2780 human ovarian cell line (40 μg/mL) (Abdel-Kader et al., 2002).
• 13*R*-*ent*-halim-1(10)-ene-15,16-diol **3** exhibited moderate activity against MDA-MB-435 (melanoma, 23 µM), SF-295 (glioblastoma, 23 µM), and HCT-8 (colon adenocarcinoma, 13 µM) was also tested as a bactericide, achieving better results (Silva et al., 2015).
• Vitextrifloxide G **5** presented potent Top1 inhibition activity, while viterofolin F **4** was much less active. Compounds **5** (20.3 µM), **7** (22 µM), and **11** (24.6 µM) showed moderate activity against HCT-116 colorectal carcinoma cells (Luo et al., 2017).
• Compound **6** presented moderate activity against AGZY 83a (lung cancer cell lines, 21.5 µM) and SMMC-7721 (liver cancer cell lines, 28.5 µM) (Yang et al., 2010).
• Vitetrifolins **7–10** showed potent to moderate cytotoxicity against the HeLa cell line (4.9–22.5 µM) (Wu et al., 2009).

The second group of natural halimanes is characterized by showing a cyclic side chain containing a furan ring (Figure 3), and all of them were tested *in vitro*, showing the following results:

• **12** exhibited moderate activity against HeLa cell lines (16 µM) (Marcos et al., 2008).
• **13** and **14** showed non-specific strong cytotoxicity against human breast ductal carcinoma (BT474), lung carcinoma (CHAGO), human liver hepatoblastoma (HEP-G2), human gastric carcinoma (KATO-3), and human colon adenocarcinoma (SW620) between 0.1 and 8.2 μg/mL (Roengsumran et al., 2004). Compound **14** inhibits K562 cell growth (9 μg/mL), while compounds **26**, **27**, and **13** are less active. These compounds were also tested against other solid tumor cell lines (>10 μg/mL) (Maslovskaya et al., 2019).
• Crassifolin F **15** showed low antiangiogenic activity (75 µM), while penduliflaworosin **16** exhibited high activity (3.4 µM) compared to the positive control (Wang et al., 2016).
• Compounds **17** and **19** were tested against PANC-1 (human ductal pancreatic carcinoma, 2.5 and 0.3 µM, respectively) and LM3 (murine lung adenocarcinoma, 3 and 40 µM, respectively) cancer cell lines, showing high cytotoxicity; while compounds **21**, **22**, and **23** were moderately active (14.1–31.6 µM) (Sánchez et al., 2010).
• Crassifolius A **18** showed cytotoxicity against Hep3B (17.9 µM) human liver cancer cell lines (Tian et al., 2017).
• Compounds **20**, **24**, **25, 36**, **37**, **38**, and **39** exhibited low to moderate cytotoxicity toward colorectal adenocarcinoma T24 (12.3–40.3 µM) and epithelial carcinoma A549 cell lines (11.6–51.9 µM) (Qiu et al., 2018).
• Chettaphanin II **28** showed cytotoxic activity against HL-60 and A549 cell lines (Yuan et al., 2017).
• Compounds **29** and **30** were tested against several cell lines but only showed moderate activity against K562 cell lines (16 and 3 μg/mL) (Maslovskaya et al., 2020).
• Crocleropenes A and B (**31** and **32**) showed weak cytotoxicity against MCF7 cell lines (36 μM and 40 µM) (Zou et al., 2020).
• Crassifolins Q and R (**33** and **34**) were tested for anti-inflammatory and anti-angiogenesis activities showing moderate activities (Li et al., 2021).
• Mallotucin D **35** was isolated in 1981, along with mallotucin C, from *Mallotus repandus* (Nakatsu et al., 1981). Although, its bioactivity was not tested until 2022, Dai et al. (2022) evaluated its activity against hepatocellular carcinoma. Mallotucin D shows the inhibition of cell proliferation and DNA synthesis plus the induction of autophagic mechanisms.

This group is characterized by a cyclic side chain consisting of a butenolide or γ-hydroxybutenolide framework (Figure 4), which shows the following result:

• *Ent*-halimanolide **40** showed cytotoxicity at micromolar levels against HeLa (5.0 µM) and MDCK (5.1 µM) cell lines (Marcos et al., 2003b).
• Echinohalimane A **41** exhibited cytotoxicity toward a variety of hematologic and solid tumor cell lines, showing better results for the latter ones including MOLT-4 (2.1 μg/mL), HL-60 (2.1 μg/mL), DLD-1 (0.96 μg/mL), and LoVo (0.56 μg/mL) cell lines (Chung et al., 2012).
• Compounds **42**, **43**, and **44** exhibited cytotoxicity toward colorectal adenocarcinoma T24 cell lines (37.3 µM, inactive, and 34.5 µM, respectively) and epithelial carcinoma A549 cell lines (18.9, 34.5, and 16.3 µM, respectively) (Qiu et al., 2018).
• Crassifolins A and B (**43**, **44**) showed moderate antiangiogenic activity (15.4 and 16.7 µM) (Wang et al., 2016).
• Compound **46** presented moderate cytotoxicity against several cell lines (13.7–16.9 μg/mL) (Scio et al., 2003).
• Compound **47**, isolated from a gorgonian coral of genus *Echinomuricea*, showed low cytotoxicity (13.2–37.1 µM) against several cell lines of hematological and solid tumors (Cheng et al., 2012).
• Crassin H **45** exhibited cytotoxic activity against HL-60 (human promyelocytic leukemia, 11.8 µM) and A549 (human lung adenocarcinoma, 5.2 µM) cell lines (Yuan et al., 2017).
• Cajucarinolide **48** and isocajucarinolide **49** are potent PLA_2_ inhibitors with IC_50_ of 5.8 and 2.3 μg/mL, respectively, (Ichihara et al., 1992). PLA_2_ may be a target in cancer treatment because it is involved in pro-inflammatory and pro-tumoral pathways (Peng et al., 2021; Vecchi et al., 2021).
• Crassifolins S-U (**50–52**) were isolated from *Croton crassifolius* and tested for anti-inflammatory and anti-angiogenesis activities (Li et al., 2021). All of them were active, and crassifolin U **52** was the most active for both bioactivities.

Finally, halimane–purine hybrids constitute this last group (Figure 5), which presented the following interesting bioactivities together with their cytotoxicity:

• Agelasine C **53** showed Na,K-ATPase inhibitory effects and antimicrobial activities (Nakamura et al., 1984).
• Agelasine J **54** presented low cytotoxicity on breast cancer MCF7 cell lines (33 µM) (Appenzeller et al., 2008).
• Agelasimines A and B (**55** and **56**) exhibited cell growth inhibition against L1210 mouse leukemia cell lines *in vitro* (ED_50_ = 2–4 μg/mL), despite their most interesting biological activity is their action as Ca^2+^ channel antagonists as well as α1 adrenergic blockers (Fathi-Afshar and Allen, 1988).
• Asmarines I and J (**57** and **58**) showed moderate cytotoxicity against a variety of cancer cell lines (Rudi et al., 2004).

## 3 Synthesis of antitumor terpenoids using *ent*-halimic acid as a starting material

In this part of the work, the synthesis of a series of antitumor compounds using *ent*-halimic acid **1** as a starting material is described.

**FIGURE 6:**
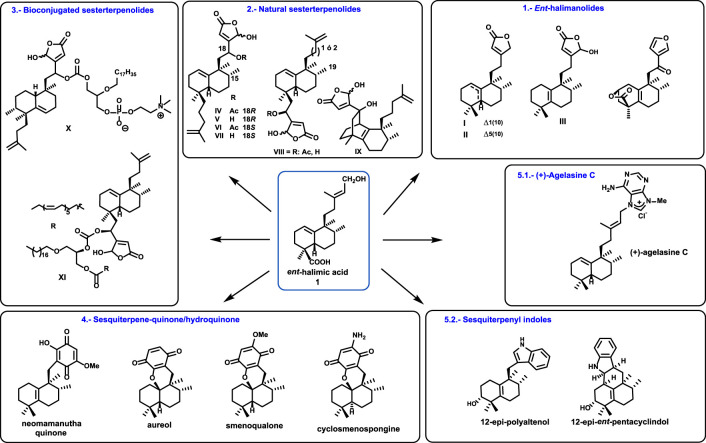
Overview of the use of *ent*-halimic acid **1**.

Using *ent*-halimic acid **1** (Figure 6) as a starting material, the following compounds have been synthesized:

1. Natural *ent*-halimanolides and furo *ent*-halimanolides.
2. Sesterterpenolides analogs of dysidiolide.
3. Sesterterpenolides hybridized with edelfosine analogs and PUFAs.
4. Quinone/hydroquinone and sesquiterpenoquinones.
5. Terpenoid alkaloids:
  5.1. (+)-Agelasine C
  5.2. Sesquiterpenyl indoles

### 3.1 Synthesis of *ent*-halimanolides

Starting from *ent-*halimic acid **1**, a series of natural *ent*-halimanolides have been synthesized. These compounds are characterized by showing a lactone ring in any position of the halimane skeleton. The synthetic routes and biological evaluations are described for each case.

#### 3.1.1 Synthesis of butenolides and γ-hydroxybutenolides

**SCHEME 1:**
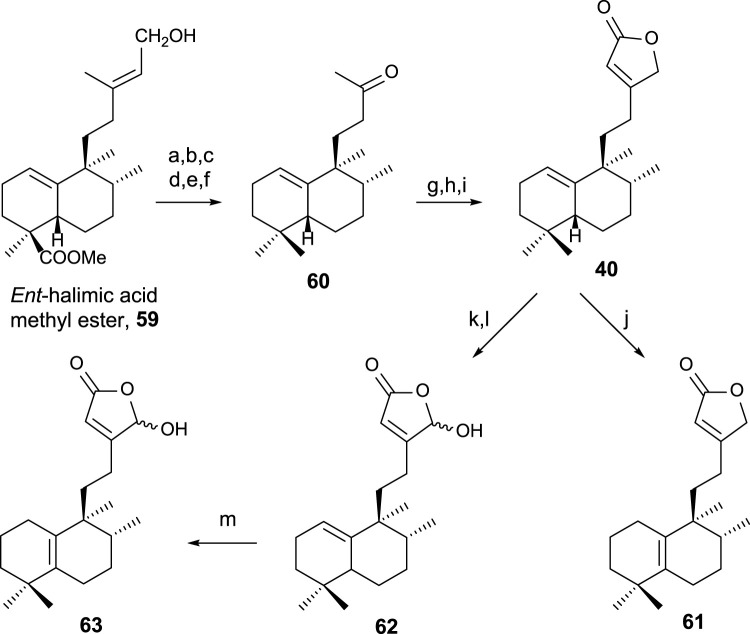
(a) NaH, MeI, and THF (92%); (b) LAH and Et_2_O (96%); (c) TPAP and NMO (91%); (d) diethylene glycol, NH_2_NH_2_·H_2_O, KOH, 175°C–230°C, and 23 h (85%); (e) *m*-CPBA (92%); (f) H_5_IO_6_, THF, and H_2_O (91%); (g) LDA, TMSCl, THF, and −78°C (97%); (h) OsO_4_, NMO, and *t*BuOH/THF/H_2_O (7:2:1) (96%); (i) Ph_3_P = C=C=O and C_6_H_6_ (91%); (j) I_2_/C_6_H_6_ (10^–2^ M) (99%); (k) LDA, TBDMSTf, THF, and −78°C (94%); (l) *m*-CPBA (77%); (m) HI/C_6_H_6_ (5 × 10^–2^ M) (99%).

The synthesis of **40**, **61**, and **63** (Hara et al., 1995) using *ent*-halimic acid methyl ester **59** as a starting material is performed according to the route showed in Scheme 1 (Marcos et al., 2003b; Marcos et al., 2005b).

The transformation of **59** of the intermediate methyl ketone **60** in six steps (Scheme 1) consists of C18 reduction and degradation of the carbon side chain. To access the key intermediate **40**, the butenolide framework is attached in three steps by a reaction with Bestmann ketene to the corresponding α-hydroxyketone of **60**, yielding **40**. For accessing **61**, the double-bond isomerization is required, and in the case of **63**, oxidation of C16 and double-bond isomerization is required (Boukouvalas and Lachance, 1998).

Compounds **40**, **61**, and **63** have been biologically evaluated, with butenolide **40** exhibiting the highest cytotoxic activity (HeLa) (Marcos et al., 2003b).

#### 3.1.2 Furo-*ent*-halimanolide synthesis

**SCHEME 2:**
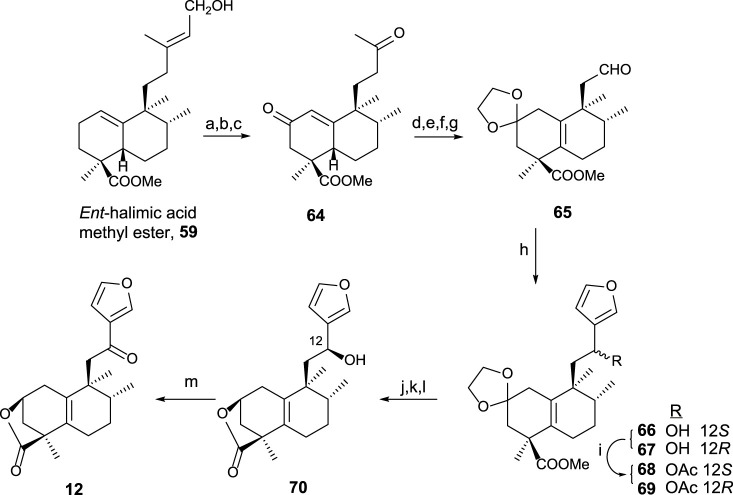
(a) OsO_4_, NMO, *t*-BuOH/THF/H_2_O (7:2:1); (b) Pb(AcO)_4_, C_6_H_6_, 20 min (94%, two steps); (c) Na_2_CrO_4_, Ac_2_O/AcOH, NaOAc, and C_6_H_6_ (64%); (d) MePPh_3_Br, NaHMDS, THF, −78°C (94%); (e) *p*-TsOH, C_6_H_6_, 60°C (96%); (f) (CH_2_OH)_2_, *p*-TsOH, C_6_H_6_, Dean–Stark (97%), (g) 1) OsO_4_, NMO, *t-*BuOH, THF, and H_2_O; 2) Pb(AcO)_4_ and C_6_H_6_ (96%, two steps); (h) 3-bromofuran, *n*-BuLi, and THF (**66**: 54% and **67**: 39%); (i) Ac_2_O and pyridine (98%); (j) HCl·2M and EtOH (96%); (k) Na_2_CO_3_, MeOH, 2 h, (96%); (l) NaBH_4_ and EtOH (12*S*: 38% and 12*R*: 43%); (m) TPAP, NMO, DCM, rt, 50 min (92%).

Using **59** as a starting material (Marcos et al., 2008), furo-*ent*-halimanolide **12** was synthesized (Scheme 2).

The synthesis of **12** from *ent-*halimic acid methyl ester **59** uses aldehyde **65** as an advanced intermediate. The transformation of **59** into **64** consists of the degradation of the side chain and C-2 functionalization, and then the tetranoraldehyde **65** is synthesized by side chain shortening and C-2 functionalization, which in parallel causes the double-bond isomerization. The furyl fragment is coupled to the aldehyde **65** in the side chain. After that, lactonization and C-12 oxidation lead to **12** in good yield.

The biological assays carried out showed antitumor activity for compound **12** against HeLa cell lines (Marcos et al., 2003b).

### 3.2 Sesterterpenolide synthesis

The study of marine metabolites has aroused great interest in recent years. Among these natural products, a considerable number of sesterterpenoids have been isolated (Faulkner, 2001; Blunt et al., 2003). Many of them possess a γ-hydroxybutenolide (Brohm and Waldmann, 1998; Demeke and Forsyth, 2000; Piers et al., 2000) moiety as a significant structural feature and, in many cases, are involved in their biological activities.

**FIGURE 7:**
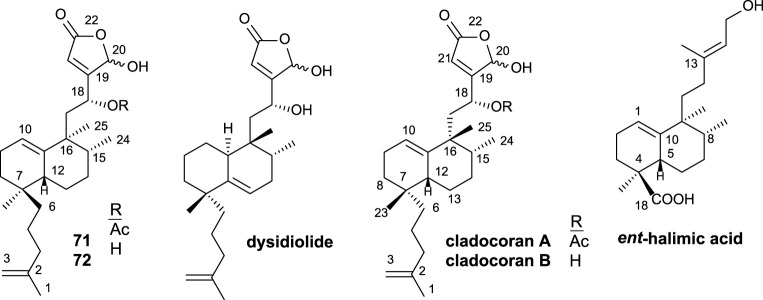
Some structures of sesterterpenolides synthesized from *ent*-halimic acid: dysidiolide and analogs, cladocoran A, and cladocoran B.

Cladocoran A and B (Figure 7) are sesterterpenolides, whose initially proposed structures were **71** and **72** (Fontana et al., 1998). The synthesis of these structures from *ent*-halimic acid, together with the spectroscopic data of the natural products named cladocoran A and B, led to the suggestion of a revision of their proposed structures as isoprenyl *ent*-halimanolides.

The synthesized isoprenyl *ent*-halimanolides **71** and **72** are dysidiolide analoges (Gunasekera et al., 1996), sesterterpenolides, that have attracted considerable attention from chemists, biologists, and pharmacologists due to their biological evaluations as antitumor agents (Corey and Roberts, 1997; Magnuson et al., 1998; Demeke and Forsyth, 2000; Eckstein, 2000; Brohm et al., 2002a).

#### 3.2.1 Synthesis of sesterterpenolides 71 and 72

Together with the synthesis of **71** and **72** (Marcos et al., 2002; Marcos et al., 2003a), the synthesis of the enantiomers and their C-18 epimers by Miyaoka et al. (2003) made it possible to establish the structure of the natural products cladocoran A and B as an olefinic analog of dysidiolide (cladocoran B) and its acetate (cladocoran A).

**SCHEME 3:**
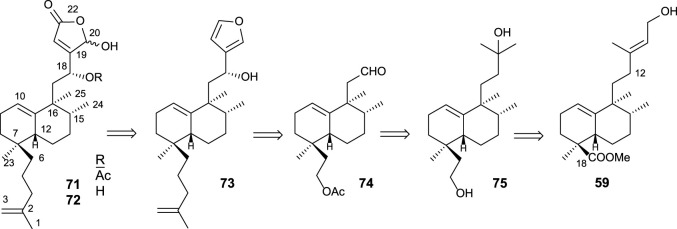
Retrosynthesis for **71** and **72** from *ent*-halimic acid.

The synthesis of **71** and **72** using **59** as a starting material was accomplished according to the following retrosynthetic pathway (Scheme 3).

**SCHEME 4:**
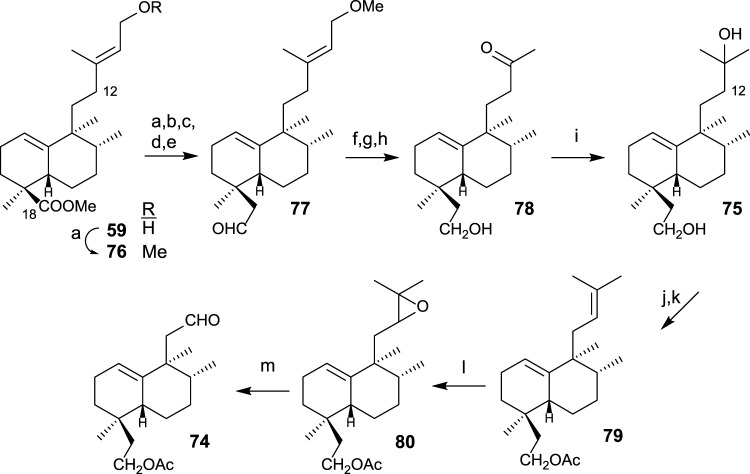
(a) LAH, Et_2_O, 1 h (96%); (b) TPAP, NMO, DCM, 15 min, (90%); (c) TsCl, pyridine, 16 h (96%); (d) (MeOCH_2_PPh_3_)^+^Cl^−^, NaHMDS, THF, −78°C, 20 min (92%); (e) acetone/H_2_O, *p*-TsOH (0.3 mol/mol), 4 h (**77**: 98%); (f) LAH, Et_2_O, 30 min (96%); (g) OsO_4_, NMO, *t*-BuOH/THF/H_2_O (7:2:1), 24 h (99%); (h) LTA, C_6_H_6_, and 20 min (95%); (i) MeMgBr, Et_2_O, −78°C, and 1 h 30 min (91%); (j) Ac_2_O, pyridine, and 5 h (95%); (k) POCl_3_, pyridine, r.t., and 1 h; (l) *m*-CPBA, DCM, 0°C to r.t., and 2 h (90%); (m) H_5_IO_6_, THF, H_2_O, and 15 min (46%).

The synthesis of diol **75** (Scheme 4) is afforded by transformation in seven steps. First, the homologation and reduction of C18 position are performed, yielding intermediate **77**, to which the side chain is degraded and adequately functionalized, leading to diol **75**. The synthesis of aldehyde **74** from intermediate diol **75** is performed in four steps consisting of the shortening of the side chain in two carbons (C13 and C16) (Scheme 4). For that purpose, the following transformations are required: protection of the primary alcohol as an acetyl derivative and dehydration of the side chain alcohol; then, oxidation and cleavage of the resulting epoxide.

**SCHEME 5:**
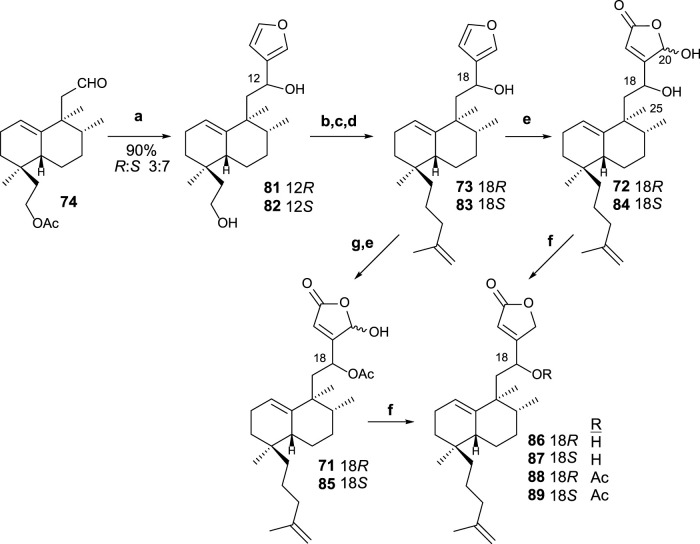
(a) 3-bromofurane, *n-*BuLi, THF, −78°C, and 20 min; (b) TsCl, pyridine, and 4 h; (c) NaI and acetone; (d) CH_2_ = C(CH_3_)-CH_2_MgCl, THF, and 12 h. (e) ^1^O_2_, hν, rose bengal, DIPEA, DCM, −78°C, and 2 h 30 min; (f) NaBH_4_, EtOH, and 10 min; (g) Ac_2_O, Pyr, and 8 h.

The reaction of aldehyde **74** with 3-furyllithium led to furo derivatives **81** and **82** (Scheme 5). Taking each C8 epimer separately, **81** and **82** are transformed in the corresponding isoprenyl-*ent-*furohalimanes **83** (18*S*) and **73** (18*R*), respectively, by elongation of the carbon chain of the southern part in three steps.

Finally, the oxidation of furo derivatives **73** and **83** led to their corresponding γ-hydroxybutenolides **72** and **84**. When C18 is first acetylated, the same procedure applied to compounds **71** and **85**. The γ-hydroxybutenolides **71**, **72**, **84**, and **85** can be decreased, respectively, to the corresponding butenolides **88**, **86**, **87**, and **89**. The interest in disposing of other synthetic analogs of dysidiolide for their antitumor activities led the obtention of these butenolide-containing sesterterpenoids, readily available from their corresponding γ-hydroxyderivatives.

The cytostatic and cytotoxic properties in tumor cell lines (HeLa, HL-60, HT-29, and A549) of compounds **72**, **84**, **85**, and **87** were determined showing IC50 in the range of 0.9–7.9 μM. The results are comparable with dysidiolide (Takahashi et al., 2000) where the synthesized compounds improve the antitumor activity against some tumor cell lines.

**FIGURE 8:**
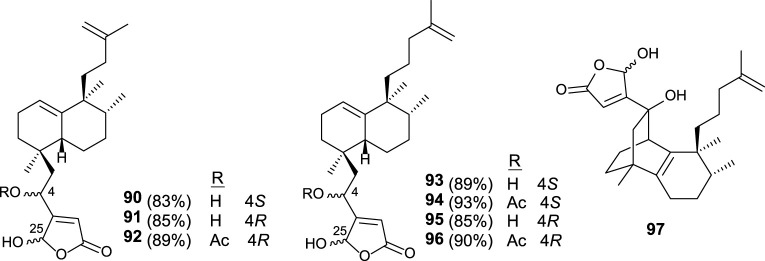
Dysidiolide analogs synthesized from *ent*-halimic acid.

Similar synthetic procedures were applied for the synthesis of new dysidiolide analogs (**90–96**), where the butenolide moiety appears in the south part and the isoprenyl unit is bonded as a carbon side chain (Figure 8). Other analogs (**97**) have been synthesized showing a tricyclic core system in which the γ-hydroxybutenolide is attached (Marcos et al., 2007).

The *in vitro* antitumor activity for these synthetic compounds was tested in HeLa, HL-60, HT-29, and A549 cell-lines. The capability of compounds **90–97** to inhibit tumor cell growth was significant in the low micromolar range. In this manner, these dysidiolide analogs are slightly more potent than proper dysidiolides (Marcos et al., 2007).

### 3.3 Synthesis of PUFAs and phospholipid hybrids with sesterterpenoids

Bioconjugate compounds have emerged in the last decades as a novel tool and therapeutic strategy in medicinal chemistry (Nilo et al., 2014; Lampkowski et al., 2015; Romero-Hernández et al., 2015; Shenvi et al., 2015; Zamudio-Vazquez et al., 2015). Bioconjugate molecules have been described as bioactive agents, showing a synergistic effect due to conjugation.

Bioconjugates of paclitaxel with polyunsaturated fatty acids (PUFAs) gave good results in anticancer therapy, reducing the toxicity and allowing a slow release in cancer cells (Bradley et al., 2001; Kuznetsova et al., 2006). Some of the most studied bioconjugates are alkyl glycerol derivatives with different biological active molecules (Huang and Szoka, 2008; Jung et al., 2008; Huang et al., 2009; Linderoth et al., 2009; Pedersen et al., 2009; Pedersen et al., 2010a; Pedersen et al., 2010b; Christensen et al., 2010; Magnusson et al., 2011; Pedersen et al., 2012). In many cases, these hybrids are considered prodrugs (Kuznetsova et al., 2006; Pedersen et al., 2009).

On the other hand, edelfosine is a well-known alkylether lipid that belongs to the so-called antitumor lipid family and is widely studied for its antitumor, antiparasitic, and other bioactivities (Mollinedo et al., 1997; Gajate and Mollinedo, 2002; Mollinedo et al., 2004). In addition, PUFAs as DHA and EPA show antitumor activity. In this way, synthesizing hybrid compounds of these antitumor molecules makes a promising approach for antitumor therapy. The following part describes the synthesis and biological results of the antitumor sesterterpenoid hybrids that are structurally related to dysidiolide with PUFAs and edelfosine analogs (Gunasekera et al., 1996; Corey and Roberts, 1997; Brohm et al., 2002a; Brohm et al., 2002b).

Taking the furyl and γ-hydroxybutenolide bioconjugates as examples for the synthesis of these kinds of compounds, the synthetic route can be divided in three steps: synthesis of the glycerolipid, conjugation with the sesterterpenoid fragment previously synthesized via carbonate linker, and finally, adequate functionalization of the glycerolipid either via phosphocholine polar head or EPA esterification.

**SCHEME 6:**
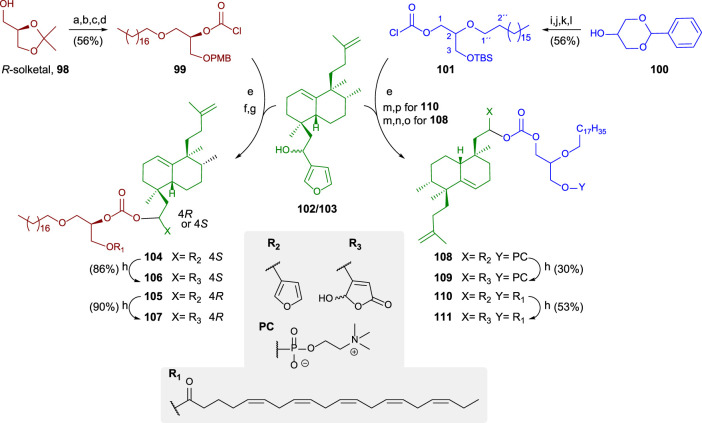
(a) Bromooctadecane, NaNH_2_, toluene, and 92%, (b) *p*-TsOH, MeOH, 40°C, and 93%, (c) 1. *n*-Bu_2_SnO and toluene, 2. CsF, PMBCl, DMF, and 80%, (d) trichloromethylchloroformate, *N,N*-dimethylaniline, THF, and 83%; (e) DMAP, DIPEA, toluene, and 60%; (f) DDQ and DCM/H_2_O; (g) EPA, EDAC, DMAP, DCM, rt, **104**: 87%, and **105**: 82%; (h) ^1^O_2_, rose bengal, DIPEA, DCM, **106**: 86%, and **108**: 90%. (i) Bromooctadecane, NaNH_2_, toluene, and 98%, (j) *p*-TsOH, MeOH, 40°C, and 90%, (k) TBDMSCl, imidazole, DMF, and rt; (l) trichloromethylchloroformate, *N,N*-dimethylaniline, THF, and 71%; (m) TBAF, THF, rt, and 89%; (n) POCl_3_, pyridine, THF, ^o^C, and 97%; (o) choline tetraphenylborate, TPS, pyridine, and 35%; (p) EPA, EDAC, DMAP, CH_2_Cl_2_, rt, and 64%.

A general synthetic scheme for the synthesis of the bioconjugates **104–111** is shown in Scheme 6 (Gil-Mesón et al., 2016). The preparation of the bioconjugate dealt here consists of three key steps: synthesis of the lipidic chain and its adequate functionalization for the hybridization step; synthesis of the sesterterpenic fragment, and finally, coupling of both fragments and derivatization for new analogs.

The glycerolipidic part of the molecule is readily prepared from *R*-solketal **100** in four steps to access the chlorocarbonate **99** that is adequate for the coupling with the sesterterpenoid **102/103** and yielding the hybrids showing the furan moiety. In these cases, the sesterterpenolide is bonded to the glycerol in *sn*2 (Gil-Mesón et al., 2016).

For preparing *sn*1-bonded sesterterpenoid from racemic glycerol, **100** is used as a starting material in the synthesis of the glycerolipid. The protected triol **100** is transformed into **101** in four steps, attaching a chlorocarbonate in *sn*1. The coupling to form the bioconjugate occurs in excellent yield. The esterification between EPA and a glycerol hydroxy group is performed to obtain the furo-bioconjugates (**104**, **105**, and **110**). The following two steps are required to attach a phosphocholine polar head to the glycerolipid: phosphorylation and choline esterification, leading to the corresponding glycerophospholipid bioconjugate (**108**). The transformation of the furylderivatives (**104, 105, 108**, and **110**) into the corresponding hydroxybutenolides can be performed by oxidation with ^1^O_2_ and rose bengal, yielding **106**, **107**, **109**, and **111**, respectively.

**FIGURE 9:**
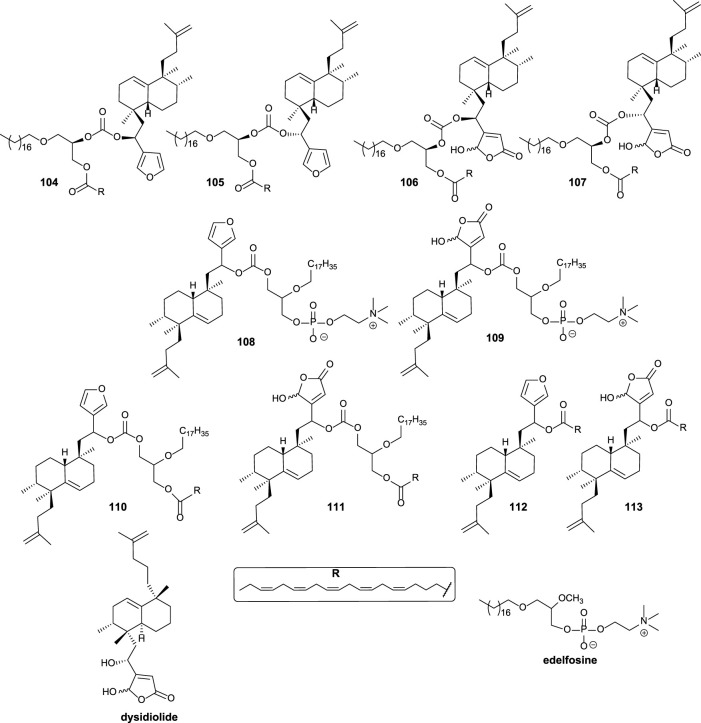
Alkyl glycerol sesterterpenoid bioconjugate compounds **104–111** and sesterterpenoid-PUFAs **112–113**, synthesized by Gil-Mesón et al. (2016).

Different structural modifications have been introduced in the synthesis of the bioconjugated compounds, including synthesizing molecules showing the furyl or butenolide fragments in the north or south part, as well as bioconjugation with a PUFA directly via a carbonate group or bioconjugation with a PUFA through a glycerol unit attached to the carbonate linker (Figure 9).

The synthesized bioconjugates have been tested against different tumor cell lines (HeLa and MCF-7). The corresponding bioconjugates showed higher antitumor activity compared with their relative non-conjugate fragments, but simple bioconjugates **112** and **113** show higher activities compared with **108** and **109**, respectively. The chirality of the glycerol unit does not seem relevant for the antitumor activity (Mollinedo et al., 1997; Samadder et al., 2004). When the γ-hydroxybutenolide moiety instead of the 3-furyl group is present in the sesterterpenoid scaffold, the antitumor activity is significantly enhanced, increasing even two orders of magnitude. For that reason, the γ-hydroxybutenolide fragments together with the bioconjugation are the highlighted structural frameworks of this family of molecules (**106**, **107**, **109**, **111**, and **113**). Although the attachment position of the sesterterpenoid fragment in glycerol does not seem very important in terms of bioactivity, when the sesterterpenoid is attached at the *sn2* position, the activity is slightly better compared to the *sn1* substitution.

### 3.4 Synthesis of sesquiterpene quinone/hydroquinone

**FIGURE 10:**
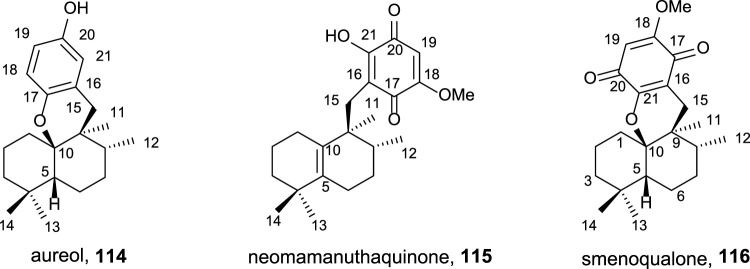
Natural sesquiterpene–quinone/hydroquinone.

The interest of these secondary metabolites (Figure 10) showing quinone/hydroquinone frameworks with drimane or rearranged drimane skeleton lies, mainly, in their biological activities and have been widely studied for this purpose (Capon, 1995; Marcos et al., 2010).

As antitumor agents, they show cytotoxicity in cancer cell lines such as HeLa, A549, HCT, KB16, P388, and Ehrlich cells, among others; moreover, antiviral, cytotoxic, hemolitic, anti-inflamatory, antiproliferative, antifeedant, antiplasmodial, antimalarial, cardiotonic antituberculosis, and antimicrobiane properties are also described. The biological assays studying the enzymatic inhibition have demonstrated the capability of different quinone/hydroquinone natural products to inhibit DNA topoisomerase I and II, tyrosine kinase, Ca^2+^/K^+^ ATPase, PI3 kinase, and PTP1B as well as interleukin-8 dissociation of its receptors.

**SCHEME 7:**
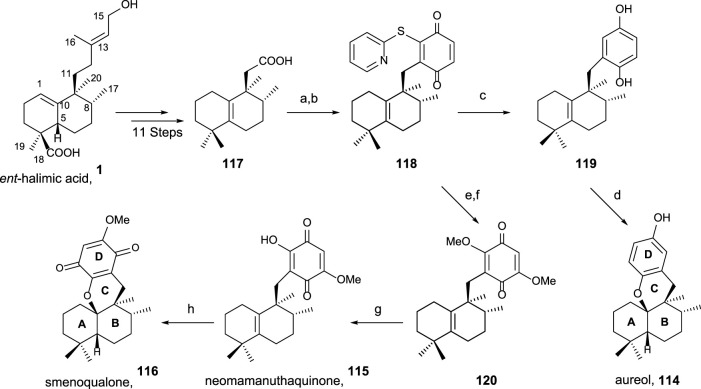
Synthesis of **114–116** from *ent*-halimic acid **1**. (a) 2-mercaptopyridine *N*-oxide, DCC, DCM, rt, 16 h, and darkness; (b) *p*-benzoquinone, DCM, hν 500W, 0°C, 2 h, and 65% from **117**; (c) Ni-Raney, EtOH, rt, 5 min, and 99%; (d) BF_3_·Et_2_O, DCM, −50°C→−5°C, 2 h, and 60%; (e) MeONa, THF, −20°C, 10 min, and 70%; (f) NaOMe, MeOH, −20°C→2°C, and 3 h; (g) HClO_4_ 60%, THF, rt, 4 h, and 70%; (h) *p*-TsOH, C_6_H_6_, reflux, and 30 min.

Using *ent*-halimic acid **1** as a starting material, different natural products (**114–116**) showing drimane or rearranged drimane skeleton have been synthesized (Talpir et al., 1994; Kurata et al., 1996; Fraga, 2003). The synthesis afforded for these compounds is shown in Scheme 7 and consists in the following key-steps: degradation of the side chain and C-18 reduction to obtain the tetranor intermediate **117**; ring-D coupling and functionalization; and finally, cyclization if needed for ring C formation.

The transformation of *ent-*halimic acid **1** into tetranorhalimane **117** takes place in 11 steps with good overall yield (Scheme 7). Thiopyridine quinone **118** is prepared by the Barton decarboxylation and rearrangement. Quinone **118** is an intermediate for the synthesis of aureol **114** and the tri and tetracyclic quinones **115** and **116**, respectively. For the synthesis of aureol **114** from **118**, two steps are required: first, the reduction to hydroquinone **119** and then the cyclization in acidic medium, accessing aureol **114** in this way. On the other hand, the addition of two equivalents of sodium methoxide in two steps yield **120** that can be transformed into neomamanuthaquinone **115** by acidic hydrolysis with HClO_4_. Finally, menoqualone **116** is synthesized from **115** by reaction with *p*-TsOH, promoting its cyclization and ring-C formation.

### 3.5 Sesqui- and diterpene alkaloids

The use of *ent*-halimic acid **1** as a starting material for sesqui- and diterpene alkaloids has been applied for the preparation of 7,9-dialkylpurines (agelasine C) as well as di- and sesquiterpenyl indoles.

#### 3.5.1 Synthesis of agelasine C

Agelasines are a diterpene alkaloid group isolated as 7,9-dialkylpurine salts from *Agelas* spp. marine sponges (Rosemeyer, 2004).

**FIGURE 11:**
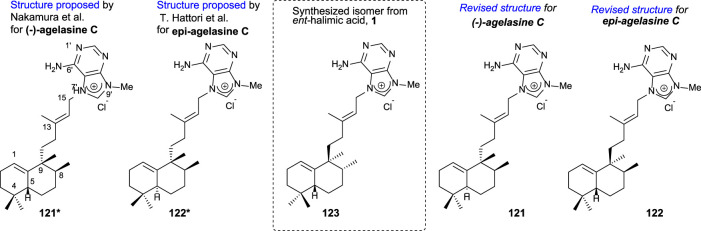
Initially proposed structures for agelasine C and epi-agelasine C as well as the revised structures corrected due to the synthesis of **123** from *ent*-halimic acid, whose absolute configurations were known.

In particular, agelasine C (Figure 11) is one of the first four known agelasines isolated by Nakamura et al. (1984) from an Okinawan sea sponge of genus *Agelas*. The biological studies of (-)-agelasine C showed a high activity inhibiting Na^+^/K^+^ ATPase and antimicrobial activities.

Later on, Hattori et al. (1997) isolated epi-agelasine C **112** from marine sponge *Agelas mauritania* as an antifouling active agent against macroalgae.

The comparison of the spectral data of compounds 121* and 122* with those of **123** suggested to correct the initially proposed structures to the revised structures (**121** and **122**) appearing in Figure 11.

**SCHEME 8:**
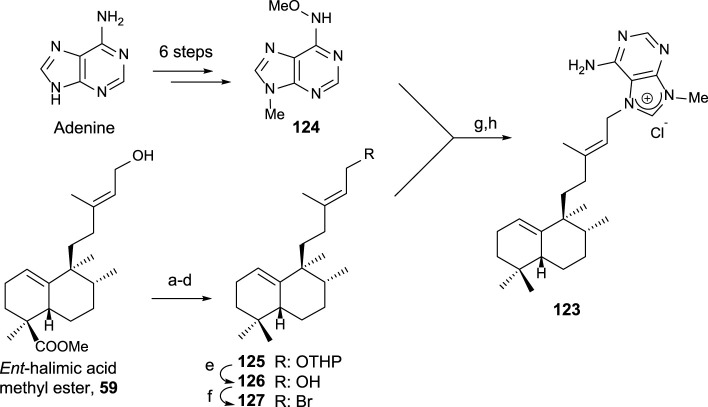
(a) DHP, p-TsOH, and C_6_H_6_ (98%); (b) LAH, Et_2_O, 0°C, and then rt (99%); (c) TPAP and NMO (94%); (d) diethylene glycol, NH_2_NH_2_·H_2_O, KOH, and 175°C–230°C (81%); (e) *p*-TsOH and MeOH (81%); (f) CBr_4_, PPh_3_, and DCM (76%); (g) DMA and 50°C; (h) Zn, MeOH, H_2_O, and AcOH (13% two steps).

Due to the interest on the biological properties of natural agelasine C and epi-agelasine C, the synthesis of **123** analog was carried out (Marcos et al., 2005a). The synthetic strategy used for accessing this meroterpenoid consists of the preparation of the diterpene fragment functionalized with bromine and then coupling with the purine derivative (Scheme 8).

#### 3.5.2 Synthesis of sesquiterpenyl indoles

Terpenyl indoles are natural meroterpenes composed of indole alkaloids and terpenoids (Marcos et al., 2013a). Among these natural products, sesquiterpenyl indoles are a small group of natural occurring products of great interest due to their variety of biological properties, such as anticancer, antibacterial, or anti-HIV (Williams et al., 2010; Marcos et al., 2013a). The first sesquiterpenyl indole described was polyalthenol **128**, isolated in 1976. After that, different natural products have been identified as sesquiterpenyl indoles, but pentacyclindole **129**, isolated from *G. suaveolens* roots*,* was a new natural product showing a novel framework. Both natural product structures, **128** and **129**, were corroborated as well as the absolute configuration determined by synthesis using *ent*-halimic acid **1** as a starting material, whose synthetic routes are described in the following section.

##### 3.5.2.1 Synthesis of 12-epi-polyalthenol

In order to confirm the structure of polyalthenol **128**, the synthesis of **130** and **131** was carried out (Marcos et al., 2012).

**SCHEME 9:**
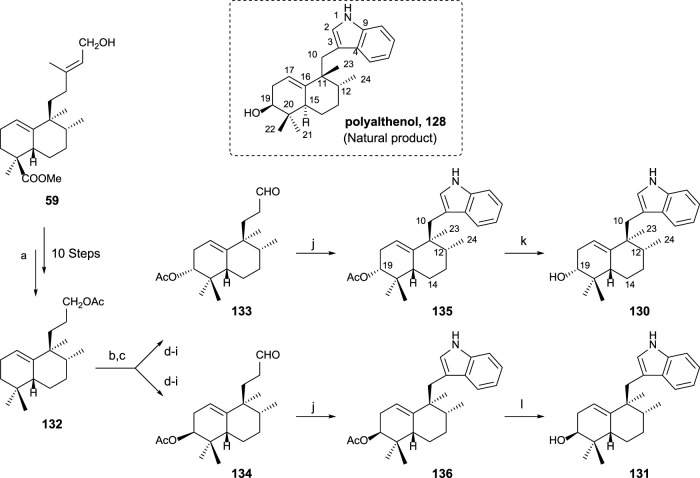
Synthesis of (-)-12-epi-polyalthenol **130** and analogs: (a) Ref Marcos et al. (2003b) and Marcos et al. (2012). (b) Na_2_CrO_4_, NaOAc, Ac_2_O, AcOH, C_6_H_6_, 55°C, and overnight; (c) Mn(OAc)_3_·2H_2_O, C_6_H_6_, and Dean–Stark 130°C; (d) 1,2-ethanedithiol, BF_3_·Et_2_O, 0°C, and overnight; (e) 10% KOH/MeOH, and 24 h; (f) Ni-Raney, EtOH, 50°C, and 1 h; (g) Ac_2_O, Py, and overnight; (h) Na_2_CO_3_ 0.7%, MeOH, 2 h, and rt; (i) TPAP, NMO, molecular sieves 4 Å, rt, and 10 min (eight steps from **132**: 10% **133** and 12% **134**); (j) phenylhydrazine, AcOH, rt, 2 h, then 130°C, 2 h, **135**: 91%, and **136**: 82%; (k) K_2_CO_3_ 3%, MeOH, rt, 24 h, and 85%; (l) 10% NaOH/MeOH, rt, 24 h, and 92%.

The synthesis of sesquiterpenyl indoles **130** and **131**, analogs of polyalthenol (Scheme 9), required the following transformations: preparation of trinorderivative **132**; C-3 functionalized intermediates **133** and **134**; and finally, indole formation (**135** and **136**) plus alkaline hydrolysis (**130** and **131**). Those transformations are presented in Scheme 9.

By the synthesis of both epimers at C19, that stereocenter was easily determined compared with polyalthenol. **130** is the one that showed a similar NMR pattern for the signal corresponding to C19. The comparison of the spectroscopic data allowed to conclude that **130** is a C12 epimer of polyalthenol. That fact, together with the opposite rotatory power shown by these molecules led to define **130** as (-)-12-epi-*ent*-polyalthenol, confirming the structure of the natural product **128** as a halimane-skeleton derivative of the normal series, and, in consequence, **130** is 12-epi-*ent-*polyalthenol.

The biological studies of **130**, **131**, **135**, and **136** were performed, where these compounds showed significant antitumor and antiproliferative activities against three cancer cell lines (A549, HL-60, and MCF-7).

##### 3.5.2.2 Synthesis of 12-epi-*ent*-pentacyclindole 137

**SCHEME 10:**
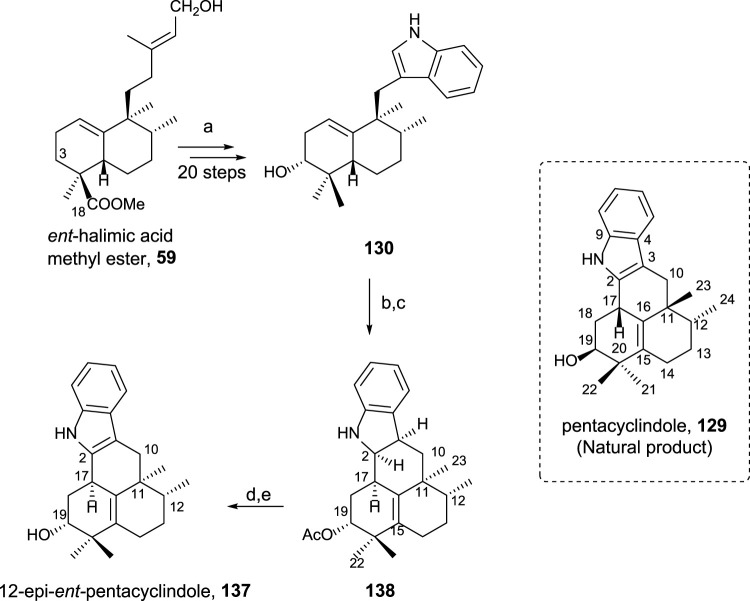
(a) Ref Marcos et al. (2012); (b) Ac_2_O, Pyr, rt, and 20 h (99%); (c) HI 57%, C_6_H_6_, 85°C, and 75 min (93%); (d) TPAP, NMO, and 4 Å molecular sieves, DCM, rt, and 15 min (66%); (e) 10% KOH/MeOH, rt, and 3 h (93%).

Pentacyclindole is a unique natural product due to its structure, being the only natural product known with its pentacyclic framework. The synthesis of its 12-epi diastereoisomer using *ent-*halimic acid uses the previously synthesized indolederivative **130** as an intermediate (Scheme 10). The cyclization bonding C-2 and C-17 in this biomimetic synthetic route can be considered the key step in the preparation of pentacyclindole analogs. Pentacyclindoles **129** and **137** are epimers at C-12, and by synthesizing **137** from *ent*-halimic acid **1**, which is carried out with excellent yields, the absolute configuration of **129** was possible to be established (Marcos et al., 2013b).

##### 3.5.2.3 Polyalthenol and pentacyclindole analogs as antitumor compounds

**FIGURE 12:**
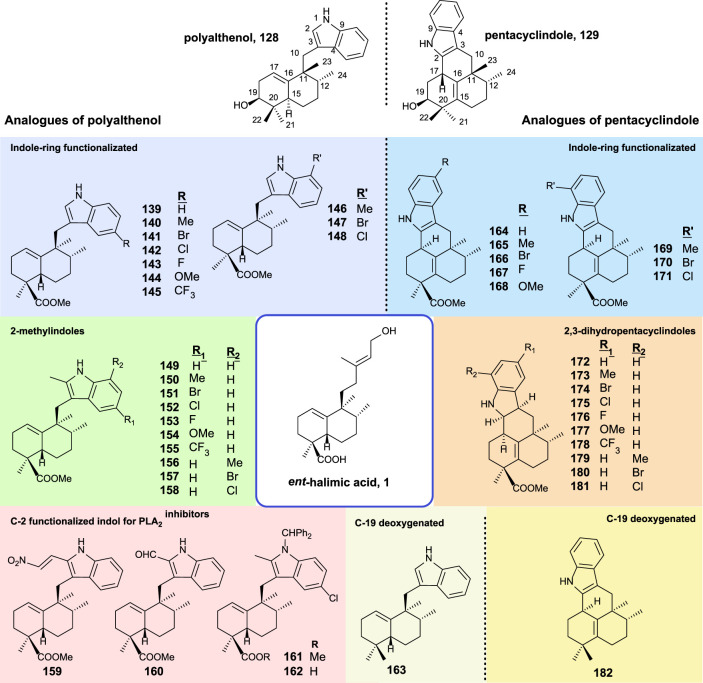
Synthesized analogs of polyalthenol **128** and pentacyclindole **129** using *ent*-halimic acid **1** as a starting material.

The synthesis and biological activity of polyalthenol and pentacyclindole analogs using similar synthetic routes, as shown for the synthesis of the epimer natural products, have been carried out (Marcos et al., 2014). A summary of all the compounds synthesized from *ent*-halimic acid **1** is displayed in Figure 12.

The structural modifications introduced in the analogs of polyalthenol can be divided into indole-ring functionalized, 2-methylindoles, C-2 functionalized, and C-19 deoxygenated (Figure 12).

In the case of the synthesis of PLA_2_ inhibitors, the most remarkable considerations are that the indole C-2 must be functionalized, and other requirements are the carboxylic acid as well as the nitrogen protected with a bulky group.

The antibacterial activity for these compounds is reported. The antiproliferative activities of these analogs have been tested in several tumor cell-lines (A549, HBL-100, HeLa, SW1573, T-47D, and WiDr). All compounds exerted inhibition of cell growth in the range of 1–70 μM. In terms of structural activity, it can be concluded from this study that the presence of substitutions in C-2 decreases the activity slightly (**139–148** vs*.* **149–158**). When substitution appears in the benzene ring of the indole either at C-6 or C-8, only the methoxy group enhances the activity (**144** and **154**). When a methyl group esterifies carboxylic acid in **161** resulting in a total loss of bioactivity, however, the free carboxylic acid in **162** recover the antitumor properties.

In the synthesis of pentacyclindole analogs, the appearing structural mosaics in the synthesized analogs of pentacyclindoles can be divided in the function of the position: indole-ring functionalized, 2,3-dihydrofuran, and C-19 deoxygenated derivatives.

For the pentacyclindole analogs, C-3 unsaturated compounds (**164–171**) always show better results in the antiproliferative experiments than the corresponding 2,3-dihydropentacyclindole derivatives (**172–181**). The presence of a methoxy group (**168**) in the benzene ring results in a little improvement of the antitumor activity in some cell lines. Compounds **168** and **164** showed the best antiproliferative activity of the pentacyclindole analogs with a GI_50_ in the micromolar range.

## 4 Conclusion

In this article, we have put together a series of natural halimanes with antitumor activity evaluated against different pharmacological targets and in different cell lines. Structurally, the halimanes that exhibit antitumor activity can be classified into four groups: halimanes with acyclic side chain, 14,15-furohalimanes, 14,15-halimanolides, and halimane–purine hybrids.

Moreover, we have reviewed the role of *ent*-halimic acid **1** as a starting material in numerous syntheses of bioactive compounds. This is a key aspect of this compound that confers it great importance, as it can be obtained easily from the extracts of *Halimium viscosum,* reducing time and effort for the obtention of more complex structures. Some of them, like the terpene–purine hybrids or the bioconjugates with antitumor lipids (edelfosine) or PUFAs, show promising results as cytotoxic and antitumor compounds, and more studies should be made to improve the bioactivities and understand the antitumor mechanism.
